# **Activation of nuclear factor-**κB **during retinal degeneration in *rd* Mice**

**Published:** 2008-06-10

**Authors:** Hui-yang Zeng, Mark O.M. Tso, Shenghan Lai, Hong Lai

**Affiliations:** 1 Peking University Eye Center, Peking University Third Hospital, Beijing, China; 2 Wilmer Eye Institute, Johns Hopkins University School of Medicine, Baltimore, MD; 3 Department of Pathology, Johns Hopkins University School of Medicine, Baltimore, MD

## Abstract

**Purpose:**

Transcription factors of the nuclear factor-kappa beta (NF–κB) family have been demonstrated to play an important role in the regulation of gene expression in the chronic neurodegenerative disorders. The aims of the current study were to investigate the alteration of NF-κB activity during retinal degeneration in *rd* mice and further explore its role in photoreceptor apoptosis.

**Methods:**

Activation of NF-κB and its nuclear translocation in the retina of *rd* mice at postnatal days (P) 8, 10, 12, 14, 16, 18, and 28 were studied by immunohistochemical analysis using NF-κB P65 antibody. The amount of NF-κB P65 protein and NF-κB DNA-binding activity in the whole retina were assessed by western blot analysis and gel shift analysis, respectively. Expression of NF-κB in microglial cells labeled with CD11b was determined by double labeling.

**Results:**

NF-κB P65 nuclear translocation and its DNA binding activity started to increase in the *rd* retina at P10 and reached a peak at P12. Expressions of P65 remained at high levels from P12 to P18. Double labeling of P65 with CD11 at P14 showed colocalization of P65 in the microglial cells in the outer nuclear layer.

**Conclusions:**

NF-κB was activated in the retinal degeneration of *rd* mice. NF-κB modulation may play a role in the retinal degeneration through microglial activation.

## Introduction

Recent studies have shown that there is a strong link between inflammation and chronic neurodegenerative diseases, such as Alzheimer disease, Parkinson disease, and Creutzfeldt–Jakob disease [1]. The common feature of these diseases is neuroinflammation, defined as the presence of activated microglia and inflammatory mediators. Microglia, resident macrophages of the central nervous system (CNS), are central to the inflammatory response. These cells actively monitor their environment and can become over-activated in response to diverse stimuli to produce cytotoxic factors, such as superoxide [2], nitric oxide [3], and tumor necrosis factor alpha (TNF-α) [4,5].

Retinitis pigmentosa (RP) is a group of inherited retinal degenerations characterized by progressive loss of photoreceptor cells. More than 158 genes causing this inherited retinal disease have been identified in about two-thirds of the cases (Retnet). However, the molecular mechanisms by which these gene mutations lead to photoreceptor apoptosis have not been clearly elucidated. In a recent study, we showed several inflammatory events and factors were involved in the retinal degenerative process of *rd* mice [6]. In this widely used retinitis pigmentosa animal model, a mutation in the gene encoding the β subunit of retinal cyclic guanosine monophosphate (cGMP) phosphodiesterase results in elevation of cGMP levels in rod photoreceptors, leading to massive cell death by apoptosis [7,8]. In that study, the activation of microglia, as well as expression of chemokines and microglia-derived neurotoxic cytokines (TNF-α), coincided with or preceded the occurrence of photoreceptor apoptosis, suggesting inflammatory response may play an important role in the retinal degeneration in *rd* mice.

The proinflammatory responses of immune cells, including microglia, involve the signal transduction molecule transcription factor nuclear factor-kappa beta (NF-κB) [9]. Activation of NF-κB contributes to microglia activation and production of proinflammatory molecules [10] that can lead to neurotoxicity in vivo [11]. NF-κB signaling begins with phosphorylation and degradation of IκB, a key component of the cytoplasmic NF-κB complex [12], and releases the p50 and p65 subunits that translocate to the nucleus and promote transcription of proinflammatory genes [13]. In many neurodegenerative diseases, a significant increase of NF-κB activity was detected in neurons and microglial cells in brains of patients [14–17]. In this study, we examined the activity of NF-κB during retinal degeneration of *rd* mice and defined its role in the inflammatory process of this neurodegenerative model.

## Methods

### Animals

This study used 120 inbred C3H/HeJ *rd* and wild-type C3H mice (Jackson Laboratories, Bar Harbor, ME) . All animals were treated in accordance with the ARVO statement for the use of Animals in Ophthalmic and Vision Research. Euthanasia was performed by placing mice in a CO_2_ chamber for 60 s, followed by cervical dislocation. The globes of the *rd* mice were collected on postnatal days (P) 8, P10, P12, P14, P16, P18, and P28. Age-matched normal C3H mice were used as controls. Mice were euthanized at the same time of the day (1:00 PM).

**Figure 1:**
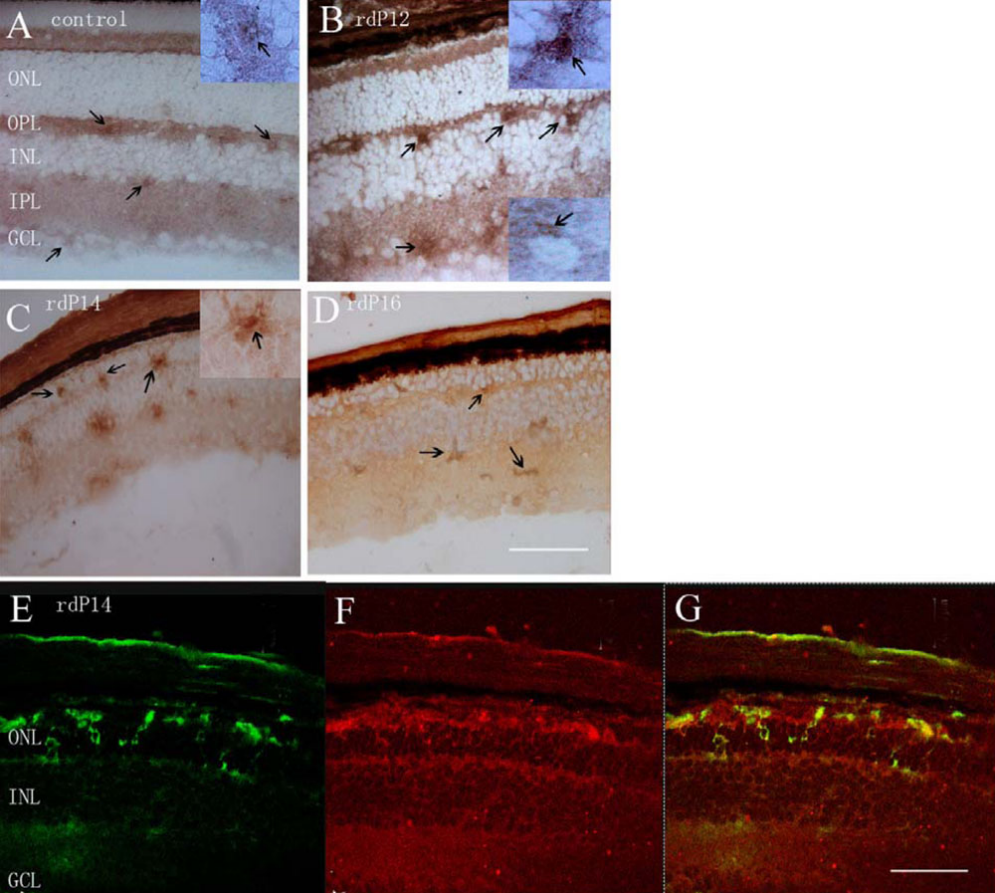
Immunochemical localization of NF-κBP65 in *rd* retinas and controls. **A:** In control retinas, weak immunoreactivity was present in the cytoplasm of cells in the ganglion cell layer (GCL), inner plexiform layer (IPL), and outer plexiform layer (OPL). The inserted picture showed magnified positive cells in the OPL. **B:** In the *rd* retina at P12, P65 immunoreactivity peaked, and prominent nuclear labeling of the cells was evident in the OPL. P65 immunoreactivity remained in the cytoplasm in the cells of GCL. The inserted picture in the upper right angle showed a magnified cell with nuclear labeling in the OPL. The inserted picture in the lower right angle showed a magnified cell with cytoplasm labeling in the GCL **C:** In the *rd* retina at P14, migration of the cells with intense nuclear labeling was seen toward the outer nuclear layer (ONL). The inserted picture showed a magnified cell with nuclear labeling in the ONL. **D:** In the *rd* retina at P16, P65 immunoreactivity was still prominent, but the cells with nuclear labeling were hardly seen. **E-G:** Double labeling of NF-κB P65 and CD 11b in the *rd* retina at P14 showed co-localization of NF-κB in microglial cells in the outer retina. **E**: Microglial cells were shown in green color; **F:** NF-κB P65 immunoreactivity was shown in red color. **G**: Expression of NF-κB P65 in the microglial cells was shown in orange color. The arrow shows positive labeling. In the figure, the inner nuclear layer is abbreviated INL. Scale bar equals 100 μm.

### Tissue preparation

After the mice were sacrificed, their eyes were rapidly enucleated and fresh-frozen in optimal cutting temperature compound (Tissue-Tek; Sakura Finetek, Tokyo, Japan) in liquid nitrogen and stored at −80 °C until sectioning. The tissue blocks were cut vertically with a cryostat at 8 μm through the optic nerve head and ora serrata.

### Immunohistochemistry of NF-κB and fluorescent double-labeling of NF-κB and microglia

NF-κB P65 immunolabeling was performed with a kit (MOM; Vector laboratories, Burlingame, CA) according to the manufacturer’s instructions. In brief, frozen sections were fixed in acetone, quenched in 0.3% H_2_O_2_ in methanol, and incubated in a mouse IgG blocking solution for 1 h. A monoclonal antibody that recognizes the active form of NF-κB (NF-κB P65; Santa Cruz Biotechnology; Santa Cruz, CA) was applied at a 1:100 dilution, and the sections were incubated at 4 °C overnight. NF-κB P65 immunoreactivity was detected using a biotinylated secondary antibody, and 3,3′-diaminobenzidine was used as the chromogen. Negative controls were performed by replacing the primary antibody with PBS.

For double labeling, the tissue sections were incubated with two primary antibodies from different species (NF-κB P65 antibody from mouse and CD 11b antibody from rat) at 4 °C overnight. After the sections were washed in PBS for 15 min, they were incubated with secondary antibodies from their corresponding species, conjugated with either 1:100 fluorescein isothiocyanate (FITC; Jackson ImmunoResearch Laboratory, West Grove; PA) or 1:200 tetramethylrhodamine isothiocyanate (TRITC) for 45 min at room temperature. The sections were observed with a confocal laser scanning microscope (TCS-NT; Leica, Wetzlar, Germany) with 488 nm filter for FITC green and 568 nm filter for TRITC. Images were captured and postprocessed by digital photography (DXL-5500; Sony, Tokyo, Japan) and confocal software (Leica).

**Figure 2:**
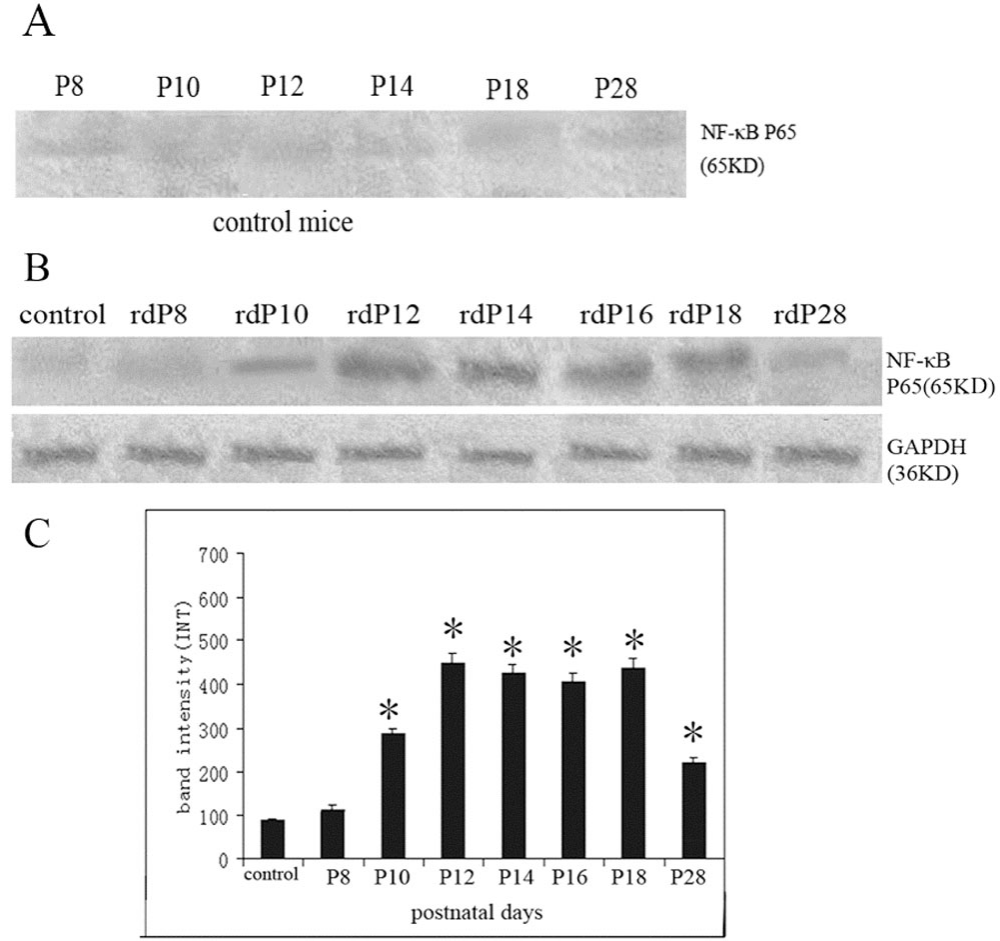
Western blot analysis of NF-κB P65 protein levels in control and *rd* retinas for each age group. **A:** Little expression of NF-κB P65 (bands at 65 kDa) was found in the control retinas at different age groups. **B:** NF-κB P65 protein was detected in the samples from *rd* retinas. GAPDH (35 kDa) was used as a loading control. **C:** Intensitometry of immunoreactive bands in *rd* retinas. Note the protein levels of NF-κB P65 in *rd* retinas were significantly increased at P10 and reached a peak at P12. It kept a high level up to P18 and was markedly reduced by P28 (*p<0.05, compared with the control retina, n=6).

### Western blot analysis

Retinal tissues were homogenized using a tissue grinder (Thomas 3431E15; Phoenix Equipment, Inc.; Rochester, NY) in 500 μl of cold suspension buffer (20 mM HEPES-KOH (pH7.5), with 250 mM sucrose, 10 mM KCL, 1 mM EDTA, 1 mM EGTA, and 1 mM DTT) containing proteinase inhibitor cocktail (Roche Diagnostics, Indianapolis, IN). The homogenates were centrifuged at 750x g at 4 °C. Each 5 μg sample was separated on a 12% SDS–PAGE gel, and western blot analysis was then performed as previously described [18]. Briefly, the electrophoresed proteins were electrophoretically transferred to nitrocellulose membrane for immunodetection. Western blots were blocked with buffer containing 5% non-fat milk and incubated with primary polyclonal antibody NF-κB P65 (sc-372; Santa Cruz Biotechnology, Santa Cruz, CA). Primary antibody binding was identified with secondary antibody conjugated to horseradish peroxidase (1:2000; Santa Cruz Biotechnology) followed by chemiluminescent detection (ECL kit, Amersham Corp., Arlington Heights, IL). The polyclonal antibody (sc-372; Santa Cruz Biotechnology), which reacts with an epitope consisting of 20 amino acids at the COOH-terminus of NF-κB P65, was used for immunodetection. Protein concentration of all samples were measured using a bicinchoninic acid (BCA) protein assay kit (Pierce, Rockford, IL) before western blot analysis, and equivalent amount of protein were used for the assay. In each blot, GAPDH was used as an internal control for the loading of protein level. Fluorescence bands were digitally captured for analysis of intensity (Quantity One Image Software; Bio-Rad Laboratories, Santa Cruz, CA).

### Electrophoretic mobility shift assay

The preparation of retinal nuclear extracts and determination of the NF-κB DNA–binding activity were performed with a nuclear and cytoplasmic reagent kit (NE-PER; Pierce) and an electrophoretic mobility shift assay (EMSA) chemiluminescence kit (LightShift; Pierce), respectively, according to the manufacturer’s protocols. A double-stranded oligonucleotide containing an NF-κB DNA–binding consensus sequence, 5′-AGT TGA GGG GAC TTT CCC AGG C-3′ (Santa Cruz Biotechnology), and a mutant double-stranded oligonucleotide, 5′-AGT TGA GGC GAC TTT CCC AGG C-3′, were used to study NF-κB DNA–binding activity, as previously described [19]. Briefly, 2 µg of nuclear extracts from the whole retina was preincubated in a reaction mixture for 20 min, and biotin end-labeled, double-stranded oligonucleotide containing the κB consensus sequence was added. Next, 5 µl of loading buffer was added to each sample. A 20 µl aliquot of the samples was electrophoresed through a 6% nondenaturing polyacrylamide gel. The hybridization signal was quantified by intensitometry using Quantity One Image Software (Bio-Rad Laboratories)

### Statistical analysis

The data were presented as mean ±standard deviation. Statistical significance was assessed with a one-way ANOVA followed by Tukey’s HSD Multiple Comparisons test. A p<0.05 was considered statistically significant.

## Results

### Nuclear translocation of NF-κB in the microglial cells in the *rd* retina

In the control retina (P14, Figure 1A), NF-κB P65 positive cells were scattered in the inner retinal layers, including the ganglion cell layer (GCL), inner plexiform layer (IPL), and outer plexiform layer (OPL). P65 immunoreactivity mainly appeared in the cytoplasm of the cells. In the *rd* retina, P65 immunoreactivity was increased and peaked at P12. At this time point, prominent nuclear translocation in the OPL was noted (Figure 1B). At P14, cells with P65 nuclear labeling were found in the outer nuclear layer (ONL; Figure 1C). At P16, P65 immunoreactivity in the retina was still prominent, but the positive cells in the outer retina were reduced (Figure 1D). Double labeling of NF-κB p65 and CD11b at P14 showed P65 immunoreactivity was predominantly in the microglial cells infiltrating the ONL (Figure 1E-G).

### Upregulation of NF-κB in the *rd* retinas

Western blot analysis was performed to assess the protein level of NF-κBP65 in retinas during retinal degeneration of *rd* mice. In the control retinas of different age groups, there was little expression of NF-κB P65 and no significant difference between each age group (Figure 2A). In the *rd* retinas, the amount of NF-κB P65 was notably increased at P10 and reached a peak at P12. The expression was kept a high level up to P18. The expression of P65 was markedly reduced at P28. (Figure 2B,C)

### Increased NF-κB DNA–binding activity in *rd* retinas

**Figure 3:**
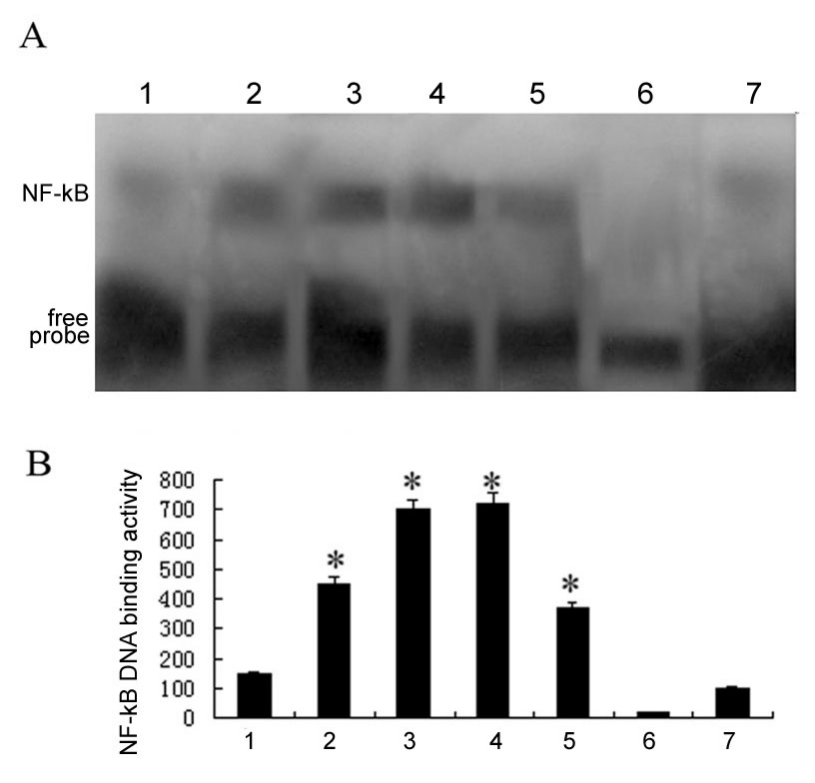
Electrophoretic mobility shift assay showing increased NF-κB DNA–binding activity in developing *rd* retinas. **A**: Representative gel shift analysis of NF-κB DNA and nuclear protein combination in control and *rd* retinas. Lane 1: NF-κB was constitutively active in control retinas. Lanes 2–5: NF-κB DNA–binding activity in the nuclei of retinal cells in *rd* mice at P10, P12, P14, and P16, respectively. Lanes 6 and 7: NF-κB DNA–binding activity by competition electrophoretic mobility shift assay (EMSA), with mutant and a hundredfold M excess of cold NF-κB oligonucleotides, respectively. **B**: Quantification of NF-κB DNA–binding activity in control and *rd* retinas shown in (**A**). Note NF-κB DNA-binding activity in *rd* retinas were significantly increased at P10, reached a peak at P12, and decreased at P16 (*p<0.05, compared with the control retina, n=6).

We performed EMSAs to determine levels of nuclear NF-κBP65 DNA–binding activity in *rd* retinas. In the EMSA blot, there were two prominent bands. The intensity of the upper band (NF-κB P65) was increased in the *rd* retina at P10, reached its maximum at P12, and decreased at P16. The specificity of the upper band was determined by the application of excess cold (unlabeled) and mutant κB double oligonucleotides in the assay. (Figure 3A,B)

## Discussion

In this study, we demonstrated increased expression of NF-κB protein and NF-κB DNA-binding activity in the retina during photoreceptor degeneration of *rd* mice. Nuclear translocation of NF-κB was noted in the microglial cells in the OPL and ONL. In a previous study [6], we showed cytokine TNF-α, a representative target and inducer gene for NF-κB activation, was also primarily produced in the activated microglial cells in the outer retina of *rd* mice. Taking our previous study and the present one together, we propose that activation of NF-κB may be associated with photoreceptor cell death through regulation of gene expression of proinflammatory and neurotoxic molecules (e.g., TNF-α) in microglial cells in the *rd* retina.

As in CNS neurodegenerative diseases, alteration of NF-κB expression has been reported in several retinal degenerations both in vitro and in vivo. Krishnamoorthy and colleagues [20] showed cultured 661W mouse photoreceptor cells constitutively expressed NF-κB, and light exposure of these cells resulted in lowering of NF-κB levels in both the nuclear and cytosolic fractions in a time-dependent manner. In contrast, Wu et al. [21]. reported in vivo expression of NF-κB in photoreceptor cells was increased at 3 h and its nuclear translocation was evident at 12 h after continuous light exposure for 3 h. Activation of NF-κB was also observed in neuronal cells in the inner nuclear layer and ganglion cell layer 24 h following retinal ischemia and reperfusion injury [22]. In the latter study, P65 labeling was partially colocalized with apoptotic cells in the inner nuclear cell layer. Consistent with the aforedescribed two animal models of retinal degeneration, our study revealed marked NF-κB activation in the retina of *rd* mice, a typical model for inherited retinal dystrophy.

In contrast to previous studies on retinal degenerations, activation of NF-κB in the *rd* retina in the present study was primarily in microglial cells, rather than in neuronal cells, including photoreceptor cells. This observation might imply a distinct function of NF-κB activation in retinal degeneration in this animal model. Pro-apoptotic and anti-apoptotic roles of NF-κB in neuronal and glial cells including microglia and astrocytes have been identified and proposed in several CNS neurodegenerative diseases [23–25]. These studies reported that activation of NF-κB in neurons played a neuroprotective role in the neurodegenerative conditions [26–28]. In this scenario, NF-κB influences the neurodegenerative process by directly promoting cell survival gene expression, such as Bcl-2, Mn-SOD, and inhibitor of apoptosis protein [29,30], or reducing cell death gene expression, such as Bxl-x and Bax in neurons [31]. In contrast, the role of NF-κB activation in glial cells, especially microglia are usually neurotoxic as production of many proinflammatory cytokines and proapoptotic substances including TNF-α, IL-1β, iNOS, and the cell adhesion molecule (ICAM-1) were regulated by NF-κB in glial cells [4,32]. In retinal degenerations, NF-κB showed a neuroprotective role in photoreceptor apoptosis in vitro as transfection of these cells with a dominant negative mutant IkB greatly enhanced kinetics of down modulation of NF- B, resulting in a faster photo-oxidative stress-induced apoptosis [20]. The function of NF-κB in the retina in vivo appeared unclear, although P65 immunolabeling positive cells were TUNEL negative in light-induced photoreceptor degeneration [21]. The present study suggests a neurotoxic role of NF-κB in photoreceptor apoptosis by not only showing its persistent high expression levels in *rd* retinas spanning from P12 to P18 when the photoreceptor cell loss took place, but also revealing its activation in microglial cells and co-occurrence of increased TNF-α production in microglial cells in a typical period (from P12 to P14) of retinal degenerations of *rd* mice [6].

NF-κB is an important signaling molecule in microglial activation. Activation of NF-κB indirectly influences neurodegenerative process by regulating gene expression in microglial cells, including pro-inflammatory cytokines TNF-α [33]. TNF-α may serve as both target and inducer gene of NF-κB activation. Like other neurodegenerative diseases, TNF-α and other NF-κB-dependent gene products may be responsible for persistent NF-κB activation and sustained chronic retinal inflammation in *rd* mice, even as the photoreceptor cells disappeared in the advanced stage.

In summary, the present study showed marked activation of NF-κB in microglial cells in the retinal degenerative process of *rd* mice. Alteration of NF-κB activity may promote photoreceptor apoptosis via initiation and perpetuation of chronic inflammation in the *rd* retina, making it an extremely attractive target for therapeutic intervention.
